# A pain killer without analgesic tolerance designed by co-targeting PSD-95-nNOS interaction and α2-containning GABA_A_Rs

**DOI:** 10.7150/thno.58364

**Published:** 2021-04-03

**Authors:** Jun Li, Lin Zhang, Chu Xu, Ying-Ying Shen, Yu-Hui Lin, Yu Zhang, Hai-Yin Wu, Lei Chang, Ying-Dong Zhang, Rong Chen, Zheng-Ping Zhang, Chun-Xia Luo, Fei Li, Dong-Ya Zhu

**Affiliations:** 1 Department of Pharmacology, School of Pharmacy, Nanjing Medical University, China.; 2 Department of Pharmacy, Nanjing First Hospital, Nanjing Medical University, China.; 3 Department of Neurology, Nanjing First Hospital, Nanjing Medical University, China.; 4 Department of Medicinal Chemistry, School of Pharmacy, Nanjing Medical University, China.; 5 Institution of Stem Cells and Neuroregeneration, Nanjing Medical University, China.; 6 Center of Drug Metabolism & Pharmacokinetics, Yantai YenePharma Co., Ltd, China.

**Keywords:** neuropathic pain, central sensitization, excitatory/inhibitory synaptic transmission, analgesic tolerance, GABA_A_ receptors

## Abstract

Overactivation of N-methyl-D-aspartate receptor (NMDAR) in the spinal cord dorsal horn (SDH) in the setting of injury represents a key mechanism of neuropathic pain. However, directly blocking NMDAR or its downstream signaling, interaction between postsynaptic density-95 (PSD-95) and neuronal nitric oxide synthase (nNOS), causes analgesic tolerance, mainly due to GABAergic disinhibition. The aim of this study is to explore the possibility of preventing analgesic tolerance through co-targeting NMDAR downstream signaling and γ-aminobutyric acid type A receptors (GABA_A_Rs).

**Methods:** Mechanical/thermal hyperalgesia were quantified to assess analgesic effects. Miniature postsynaptic currents were tested by patch-clamp recording to evaluate synaptic transmission in the SDH. GABA-evoked currents were tested on HEK293 cells expressing different subtypes of recombinant GABA_A_Rs to assess the selectivity of (+)-borneol and ZL006-05. The expression of α2 and α3 subunits of GABA_A_Rs and BDNF, and nNOS-PSD-95 complex levels were analyzed by western blotting and coimmunoprecipitation respectively. Open field test, rotarod test and Morris water maze task were conducted to evaluate the side-effect of ZL006-05.

**Results:** (+)-Borneol selectively potentiated α2- and α3-containing GABA_A_Rs and prevented the disinhibition of laminae I excitatory neurons in the SDH and analgesic tolerance caused by chronic use of ZL006, a nNOS-PSD-95 blocker. A dual-target compound ZL006-05 produced by linking ZL006 and (+)-borneol through an ester bond blocked nNOS-PSD-95 interaction and potentiated α2-containing GABA_A_R selectively. Chronic use of ZL006-05 did not produce analgesic tolerance and unwanted side effects.

**Conclusion:** By targeting nNOS-PSD-95 interaction and α2-containing GABA_A_R simultaneously, chronic use of ZL006-05 can avoid analgesic tolerance and unwanted side effects. Therefore, we offer a novel candidate drug without analgesic tolerance for treating neuropathic pain.

## Introduction

Neuropathic pain can result from various conditions, including postherpetic neuralgia, painful diabetic polyneuropathy, trigeminal neuralgia, post-surgery neuropathic pain, multiple sclerosis, HIV infection, spinal cord injury, stroke and cancer, is usually severe and persistent 1-3. A best estimate of population prevalence of pain with neuropathic characteristics is likely to lie between 6.9% and 10% 4. Despite a lot of nonprescription analgesics being advertised and sold in drugstores, the treatment of neuropathic pain is still dominated by two classical medications: opioids and nonsteroidal anti-inflammatory drugs, and often fail to provide adequate long-term relief owing to analgesic tolerance and side effects.

Neuropathic pain is associated with an imbalance of activity in pain neural circuit, in which, peripheral nociceptors project onto second-order nociceptive neurons in the spinal dorsal horn (SDH), after integration and processing, net output from spinal networks is carried by several pathways to distinct projection sites in the brain 5, 6. Dorsal horn nociceptive neurons are the pivot of pain neural circuit 6, 7. Central sensitization at spinal level, a prolonged hyperexcitability of dorsal horn nociceptive neurons caused by neural plasticity, is crucial for both the development and maintenance of chronic neuropathic pain 5, 8-11.

Overactivation of quiescent N-methyl-D-aspartate receptors (NMDARs) in the SDH in the setting of injury represents a key mechanism of central sensitization 5. NMDARs antagonists (e.g. ketamine, MK801 and memantine) can produce antinociceptive efficacy in various animal pain models and in clinical practice 12-14. However, as NMDARs are involved in a wide range of physiological processes, especially in the central nervous system, directly blocking NMDA receptors will produce many severe side effects, such as cognitive dysfunction, mental disorders and motor impairment 15, which limits the clinical use of NMDAR antagonists 13, 16, 17. So, inhibiting downstream signals of NMDARs activation, such as the association of NR2B subunit of NMDARs with scaffolding protein postsynaptic density-95 (PSD-95) or the interaction between neuronal NO synthase (nNOS) and PSD-95, has become a novel strategy to treat neuropathic pain while avoiding undesirable effects of directly blocking NMDARs function 16, 17. Dissociating PSD-95 from NR2B by Tat-NR2B9c produces antinociceptive effect in neuropathic pain 18. IC87201 and ZL006, small molecular disruptors of PSD-95-nNOS interaction, also attenuate both thermal hyperalgesia and mechanical allodynia in various animal pain models 12, 17, 19, 20. Unfortunately, however, our previous study has shown that both MK801 and ZL006 cause obvious analgesic tolerance in the treatment of neuropathic pain due to GABAergic disinhibition, when used chronically 21.

As we know, loss of GABAergic inhibitory controls in the SDH also leads to central sensitization 5, 6, 22. GABAergic axons densely innervate lamina I projection neurons to decrease the excitability of lamina I output neurons in the SDH and modulate pain transmission 23. Drugs potentiating γ-aminobutyric acid type A receptors (GABA_A_Rs), such as benzodiazepines (BZDs), have been proposed as potent analgesics in various models of inflammatory and neuropathic pain. It is known that there are 4 α subunits in the mammalian brain, in which, α1-containing GABA_A_Rs contribute to BZDs-associated side effects, whereas GABA_A_Rs containing α2 and α3 subunits are critical components of spinal pain control 24, 25. Specifically targeting α2- or α3-containing GABA_A_R is highly effective against neuropathic pain and can avoid unwanted side effects 25, 26. Based on these, co-targeting PSD-95-nNOS and α2-containning GABA_A_R may develop potent pain killer without analgesic tolerance and unwanted side effects.

## Materials and Methods

### Experimental Animals

Adult male Sprague Dawley (250-300g) rats (from SIPPR-BK, Shanghai, China), adult (6- to 7-week-old) male homozygous nNOS-deficient mice (B6; 129S4-Nos1*^tm1Plh^*, Nos1^-/-^, stock number: 002633) and their wild-type controls of similar genetic background (B6129SF2, WT) (both from Jackson Laboratories; maintained at Model Animal Research Center of Nanjing University, Nanjing, China), and male young adult (6- to 7-week-old) C57BL/6 mice (from Model Animal Research Center of Nanjing University, China) were used in this study. The animals were maintained at a controlled temperature (20 ± 2 °C) and group housed (12-h light/dark cycle) with access to food and water *ad libitum*. All procedures involving the use of animals were approved by the Institutional Animal Care and Use Committee of Nanjing Medical University. Every effort was made to minimize the number of animals used and their suffering.

### Neuropathic pain model

Neuropathic pain was induced by segmental spinal nerve ligation (SNL). Animals were anesthetized with isoflurane. After the loss of righting reflex, the animal was fixed in the prone position. The SNL model was prepared as described previously 27: a median skin incision with about 3-5 cm length in L4-S2 level of the mouse back was made, the muscles next to the vertebrae till the sixth lumbar protruding were separated, the L5/L6 joint protruding on the right side was exposed and excised, and L6 processus transversus was partially split so that the L4-L6 spinal nerves on the right side were exposed. L5 nerve was gently isolated and tightly ligated with 5-0 silk thread. The wound was closed with 4-0 silk thread suture and covered with iodine solution. After nerve ligation, animals with signs of paw clonus or autotomy were excluded from further experiments.

### Drugs and their administrations

ZL006 and ZL006-05 was synthesized in our laboratory 15. (+)-Borneol (Cat#68878), diazepam (Cat#M907), MK801 (Cat#M107) and were purchased from Sigma-Aldrich. ZL006 was dissolved as described previously 15. (+)-Borneol was dissolved in Tween 80 (final concentration ≤ 3%) plus saline 28, ZL006-05, ZL006-05A and ZL006-05B was dissolved in saline containing 2% dimethylacetamide and 2% solutol ®HS 15. MK801 was dissolved in saline.

#### Continuous intrathecal infusions

Osmotic minipumps (models 1004 for mouse and 2ML4 for rat; Alzet) and intrathecal cannulas (0.61 mm outer diameter and 0.28 mm inner diameter for mouse, 0.99 mm outer diameter and 0.58 mm inner diameter for rat) were filled with a solution of PBS. Cannulas were inserted into the subarachnoid space through the space between the L5 and L6 vertebrae and the tip of the catheter was implanted at the L5 spinal segmental level 29.

### Behavioral testing

#### Mechanical nociception assays

For rats, the threshold for 50% paw withdrawal (50% PWT) response to mechanical stimulus was assessed through a dynamic plantar apparatus (Ugo Basile, Italy). The 50% PWT refers to that the probability of paw withdrawal response to repeated mechanical stimulus is 50%. Rats were placed individually in a plastic cage (17 × 15 × 15 cm) with a wire mesh bottom which allowed full access to the paws. Behavioral accommodation was allowed for 15-20 min until cage exploration and major grooming activities ceased. A maximal cut-off of 50g/50s was set to prevent tissue damage. To record the PWT in response to mechanical stimulus, each rat was measured five times with a 5-min interval, and the mean value was recorded after excluding maximal and minimum values. The experimental procedures were performed in a double-blind manner.

For mice, the threshold for 50% PWT response to mechanical stimulus was quantified by assessing the paw withdrawal threshold using von Frey filaments (Touch-Test TM Sensory Evaluator, North coast Medical, Inc.). Mice were placed individually in a plastic cage (4.5 × 5 × 11 cm) with a wire mesh bottom which allowed full access to the paws. Behavioral accommodation was allowed for 20-30 min until cage exploration and major grooming activities ceased. Mechanical threshold was measured by applying a von Frey filament to the ventral surface of the right hind paw until a positive sign of pain behavior was elicited. The paradigm for assessing the threshold was as follows. Von Frey filaments with logarithmically incremental stiffness (0.02-2 g) were applied serially to the paw by the up-down method 30. The filaments were presented, in ascending order of strength, perpendicular to the plantar surface with sufficient force to causes light bending against the paw and held for 4s. A positive response was noted if the paw was sharply withdrawn. Flinching immediately upon removal of the hair was also considered a positive response. The 2-g filament was selected as the upper limit cut-off for testing. If there was no response at 2-g filament, animals were assigned this cut-off value. The pattern of positive and negative withdrawal responses was converted to 50% threshold according to the formula: 50% PWT = 10 log (X) + κδ. X = value (in log unit) of the final von Frey hair used; κ is correction factors based on the pattern of responses from the calibration table; δ = mean difference in log units between stimuli (here, 0.224).

#### Thermal nociception assays

Paw withdrawal latency (PWL) response to thermal stimulus was assessed through the Hargreaves test. In the test, infrared heat was applied with a Hargreaves apparatus (Ugo Basile, Italy) to the plantar surface of the injured hind paw. Rats were placed individually in a plastic box (17 cm×15 cm ×15 cm) on a glass platform. Behavioral accommodation was allowed for 15-20 min. Radiant heat was shone on the center of the paws and a maximal cut-off of 30s was set to prevent tissue damage. To record the PWL in response to thermal stimulus, each animal was measured five times with a 5-min interval, and the mean value was recorded after excluding maximal and minimum values. The experimental procedures were performed in a double-blind manner.

#### Open field test

The locomotor activity was assessed by the open field test as described previously 31. The apparatus consisted of a large area composed of plastic, surrounded by walls that were 100 cm high. The floor was 50 × 50 cm for mice; the overall illumination was 25 lux. Each animal was gently placed in the center of the open field, and its behavior was videotaped. The distance the animals travelled was measured in a 5-min session using ANY-maze (Stoelting Co., IL, USA). After each trial, the plate was cleaned with 70% EtOH. No stressor was applied to the animals for at least 12 h before the test.

#### Rotarod Test

The motor function was assessed by using a RotaRod (Med Associates) 1 hour after drug administration as described previously 28. The RotaRod test was performed by measuring the time each mouse was able to maintain its balance while walking on the rotating drum. One hour before testing, mice were trained on the RotaRod at a constant acceleration of 16 rpm until they could stay on for 30 s. For testing, the RotaRod was set to accelerate from 4 to 40 rpm over a 5-min period. Mice were given three trials with a maximum time of 300 s and a 5-min intertrial rest interval. The latency to fall was recorded as a measure of motor function.

#### Morris water maze task

The Morris water maze task was used to exam spatial cognitive performance of rats. A circular swimming pool (Jiliang Neuroscience Inc.) measuring 180 cm in diameter and 48 cm in height was filled with water to a depth of 26 cm at 24 ± 2 °C. Four starting points around the edge of the pool were designated as N, E, S, and W, which divided the pool into four quadrants. A platform, 10 cm in diameter, was located in a constant position in the middle of one quadrant. To render it invisible to the rats, platform was submerged 2.0 cm below the surface of the water. The task for the rats was to escape from the water by locating the hidden platform. Two days before training, the animals were habituated to swimming for 60 s in the pool without a platform. In training session, rats were trained to escape from the water by locating the platform which was above the water with a red flag on it. Then, one block of four trails was given for 6 consecutive days, and on each day, two groups of rats were administrated with MK801 (0.25 mg/kg, i.v.) or vehicle (6 mL/kg, i.v.) 30 minutes before the first trail. Meanwhile, another two groups of rats were treated with ZL006-05 (90 mg/kg, i.g.) or vehicle (6 mL/kg, i.g.) 1 hour before the first trail. For each trial, the rat was placed in the water facing the wall of the pool at one of four starting points and allowed to swim for a maximum of 60 s. If the rats found the platform, they were remained on it for 10 s; the rat not finding the platform were guided to it and allowed to remain there for 10 s. Each trial was videotaped via a ceiling-mounted video camera and the animal's movement was tracked using Ethovision software (Noldus Information Technology, Wageningen, the Netherlands), which allows the calculation of various measures such as latency (time to reach the platform) and swimming speed. On day 7, rats were given one 60-s retention probe test in which the platform was removed from the pool. During retention, the number of crossings of the platform location and the time spent in the target quadrant were measured.

### Recombinant GABA_A_Rs in HEK293 cells

The affinities of drugs for α_1_, α_2_, α_3_ and α_5_ subunits were studied in the HEK293 cells (from FuHeng Biology, Shanghai, China) transiently expressing recombinant GABA_A_Rs (α_1_β_2_γ_2_, α_2_β_3_γ_2_, α_3_β_3_γ_2_ and α_5_β_2_γ_2_) as described previously 26. Briefly, the HEK293 cells were cultured in MEM medium supplemented with 10% FBS (fetal bovine serum, Gibico), 1% L-glutamine (Gibco) and 1% mycillin (Gibco). Before transfection, the cells were subcultured onto a 3.5cm dish at the ratio of 1:2. Then, the plasmids of α_1_/α_2_/α_3_/α_5_ (Origene), β_2_/β_3_ (Origene) and γ_2_ (Shanghai Genechem Co.,Ltd ) (5μg total DNA, ratio 1:1:2) were transfected using Fugene^®^HD transfection reagent (Promega). The γ_2_ subunit contained GFP from IRES (internal ribosomal entry site) and was used as a marker of the successful transfection. Cells were used for electrophysiology recording 18-36 hours after transfection.

### Electrophysiology

#### Electrophysiology recordings from acute brain cortex or spinal cord slices

After anesthetized by ethyl ether, rats were transcardially perfused with ice-cold cutting solution (in mM, Choline Chloride 110.0, Glucose 20.0, KCl 2.5, CaCl_2_ 0.5, MgCl_2_ 7.0, NaH_2_PO_4_ 1.3, NaHCO_3_ 25.0, Na-ascorbate 1.3, Na-pyruvate 0.6) and decapitated. Lumbar spinal cord (L4 and L5) was rapidly removed, and 350 μm cortical slices or 300 μm transverse spinal cord (embedded in a 3% agarose block) slices were made in ice-cold cutting solution using a vibratome (VT1000s, Leica). Whole-cell recordings from slices were performed at 30 °C with standard intrapipette solution (in mM: Cs-gluconate 132.5, CsCl 17.5, MgCl_2_ 2.0, EGTA 0.5, HEPES 10.0, ATP 4.0, QX-314 5.0) in normal ACSF artificial cerebrospinal fluid (in mM: glucose 10.0, NaCl 125, KCl 2.5, CaCl_2_ 2, MgCl_2_ 1.3, NaH_2_PO_4_ 1.3, NaHCO_3_ 25.0, Na-ascorbate 1.3, Na-pyruvate 0.6). All solutions were bubbled with 95% O_2_ -5% CO_2_.

For GABA-evoked currents recording, the neurons of brain cortex or spinal cord slices were viewed under upright microscopy (Olympus X51W, Nomasky), standard intrapipette solution (in mM: Cs-gluconate 132.5, CsCl 17.5, MgCl_2_ 2.0, EGTA 0.5, HEPES 10.0, ATP 4.0, QX-314 5.0) was used, and GABA was applied to the recorded cell using a manually controlled pulse (4-6s) of a low subsaturating GABA concentration. Drugs dissolved in DMSO and subsequently diluted with recording solution were co-applied together with GABA without preincubation. Recordings in which access resistance or capacitance changed by 20% during the experiment were excluded from data analysis. One neuron was recorded per slice, and no more than five slices were recorded per rat. Each set of experiments was repeated using at least 4 rats. Data were acquired using a Multiclamp 700B amplifier and filtered during acquisition with a low-pass filter set at 2 kHz and sampled at 10 kHz using pClamp 10.3 (Molecular Devices). Data analysis was performed offline with Clampfit 10.2 (Molecular Devices).

For mPSCs measurements, whole-cell recordings were made from putative excitatory neurons in superficial dorsal horn using low-resistance pipettes (4-8 MΩ). Before mPSCs measurements, excitatory neurons were distinguished from inhibitory neurons by firing pattern analysis according to the established relationship between neuronal firing pattern and its neurotransmitter phenotype 21, 32-34 For mEPSCs measurement, the membrane potential was held at -60 mV. Intrapipette solution containing Cs-gluconate 32.5, CsCl 17.5, MgCl2 2.0, EGTA 0.5, HEPES 10.0, ATP 4.0 and QX-314 5.0 (in mM) was used. Tetrodotoxin (0.5 μM) and bicuculline (20 μM) were added to block action potentials and GABA_A_ receptor-mediated currents respectively. For mIPSCs measurement, the membrane potential was held at -70 mV, microelectrodes were filled with internal pipette solution containing CsCl 140, HEPES 10.0, MgATP 4.0, NaGTP 3.0, EGTA 1.0, MgCl_2_ 1.0, CaCl_2_ 0.3 (in mM). To isolate mIPSCs, Tetrodotoxin (0.5 μM), NBQX (10μM) and APV (50μM) were in presence. Recordings in which access resistance or capacitance changed by 20% during the experiment were excluded from data analysis. One neuron was recorded per slice, and no more than five slices were recorded per rat. Each set of experiments was repeated using at least 4 animals. Each recording lasts more than 5 min. Data were analyzed using Mini software. Up to 120 events from each neuron were selected at a fixed sampling interval to generate cumulative probability. Data were acquired using a Multiclamp 700B amplifier and filtered during acquisition with a low-pass filter set at 2 kHz and sampled at 10 kHz using pClamp 10.3 (Molecular Devices).

#### Electrophysiology recordings from HEK293 cells

Electrophysiology recordings from HEK293 cells were performed as described previously 26. Briefly, whole-cell patch-clamp recordings of GABA-evoked currents were made at 30 °C with the holding potential of -60 mV. The internal solution (solution in recording electrodes) contained (in mM): 120 CsCl, 10 EGTA, 10 HEPES, 4 MgCl_2_, 0.5 GTP, 2 ATP and pH was adjusted to 7.4 with CsOH and the external solution contained (in mM): 150 NaCl, 10 KCl, 2 CaCl_2_, 1 MgCl, 10 HEPES, 10 Glucose and pH was adjusted to 7.4 with NaOH. ACSF was bubbled continuously with carbogen (95%O_2_ and 5%CO_2_). For the recording of GABA-evoked currents, the slides were transferred to a recording chamber, which was perfused with ACSF at a rate of 4 ml/min. The cells were viewed under upright microscopy (Olympus), and GFP-positive cells were selected to record the currents. The interval time between each different dose of GABA was controlled, 3 to 5 minutes to avoid the desensitization of GABA_A_Rs. Data were acquired using a Multiclamp 700B amplifier and filtered during acquisition with a low-pass filter set at 2 kHz and sampled at 10 kHz using pClamp 10 (Molecular Devices). Data analysis was performed offline with Clampfit 10.2 (Molecular Devices).

#### Estimation of EC_50_ and E_max_

EC_50_ and E_max_ values were estimated by fitting concentration-response curves to the Hill equation according to the following formula I = I_max_/(1+ [EC_50_/(A)]^h^) as described previously 35. Here, I represents the peak current at a given concentration of agonist A, I_max_ is the maximum current, EC_50_ is the concentration of agonist yielding a half maximal current, and h is the Hill coefficient. Data analysis was performed offline with Clampfit 10.2 (Molecular Devices) and OriginPro 9.4 (OriginLab).

### Tissue isolation and western blot analysis

Western bolt analysis was performed as described previously 36. Briefly, the dorsal spinal cord (L4 and L5) was dissected from sham or SNL rats and homogenized in 400μl RIPA lysis buffer containing 1 μM pepstatin, 10 μg/ml aprotinin, 10 μg/ml leupeptin, 10 μg/ml and 10 μM phenylmethylsufonyl fluoride (PMSF). After lysis for 30 min in ice, samples were centrifuged at 12,000 g for 15 min. The protein content in each supernatant fraction was determined by using Bradford's solution, and samples containing equivalent amounts of protein were applied to 10% or 15% (10% for GABA(A)α2 and α3, 15% for BDNF) acrylamide denaturing gels (SDS-PAGE). The separated proteins were transferred onto a polyvinylidene fluoride membrane. The primary antibodies were as follows: rabbit anti-GABA(A)α2 (1:500; Millipore AB5948), rabbit anti-GABA(A)α3 (1:500; Millipore AB5594), rabbit anti-BDNF (1:1000; Thermo PA1-18357, RRID:AB_1070634). Internal control was performed using rabbit anti-β-actin (1:1000; Bioss bs-0061R, RRID: AB_10855480). Appropriate horseradish peroxidase-linked secondary antibodies were used for detection by enhanced chemiluminescence (Bio-Rad).

### Coimmunoprecipitation

Lysis and coimmunoprecipitation of tissues were performed as we described previously 36. Briefly, dorsal spinal cord tissues (L4 and L5) were lysed in 400 μl RIPA containing 1 μM pepstatin, 10 μg/ml aprotinin, 10 μg/ml leupeptin, 10 μg/ml and 10 μM PMSF. The lysates were centrifuged at 12,000 × g for 15 min at 4°C. The supernatant (200 μl) were incubated with rabbit anti-PSD95 (1:40; Cell Signaling Technology 2507, RRID:AB_561221) or rabbit anti-IgG (1:40; Millipore 401590) for 8-10 h. Protein G-Sepharose beads (Sigma P4691) and immune complexes were added for incubation overnight at 4 °C. Immune complexes were isolated by centrifugation, washed five times with 0.05 M HEPES buffer, pH 7.1, containing 0.15% Triton X-100, 0.15 M NaCl, and 0.1 × 10^-3^ M sodium or thovanadate, and bound proteins were eluted by heating at 100°C in loading buffer. Proteins were analyzed by immunoblotting using rabbit anti-PSD95 (1:500; Cell Signaling Technology 3450, RRID: AB_2292883) or rabbit anti-nNOS (1:400; Thermo 61-7000, RRID: AB_2313734).

### Identification and characterization of metabolites of ZL006-05 in plasma

ZL006-05 was given to rats by intravenous injection (i.v., 6.0 mg/kg) and oral administration (i.g., 90 mg/kg) respectively. Plasma samples were collected at pre-dose and 0.25, 0.5, 2 and 4 h for i.v. and 0.5, 1, 2 and 4 h for i.g after a single dose administration. Pre-dose plasma samples were used as control blank. After vortex and centrifugation, supernatant was removed and dried out by nitrogen blowing instrument at 40 °C and then re-dissolved with 100 μL water/ACN (50/50, v/v). The resulting samples were injected and analyzed by UPLC-UV/Q-TOF MS. Metabolites were separated with ACQUITY UPLC HSS T3 C18 column (2.1 × 100 mm I.D., 1.7 µm I.D.) with column temperature at 45ºC and flow rate at 0.4 mL/min, detected by UV (200-600 nm). Masslynx V4.1 of Waters was used for data acquiring, MetaboLynx XS with Masslynx V4.1 was used for data analysis.

### Quantification and statistical analysis

All data are presented as means ± s.e.m. Comparisons among multiple groups were made with one-way ANOVA (one factor) followed by Scheffe´ *post hoc* test or two-way repeated measures ANOVA (two factors) followed by Bonferroni´* post hoc test*. Comparisons between two groups were made with a two-tailed Student's *t test*. Statistical significance was set at *p* < 0.05. The sample size was predetermined by analyzing pre-experimental data with PASS (power analysis and sample size) software. For animal studies, the sample size was predetermined by our prior experience. Randomization was used in all experiments. Investigators were blind to treatment group when assessing the outcome.

## Results

### Prolonged disassociation of PSD-95-nNOS by ZL006 produces analgesic tolerance and induces disinhibition

**Figure 1:**
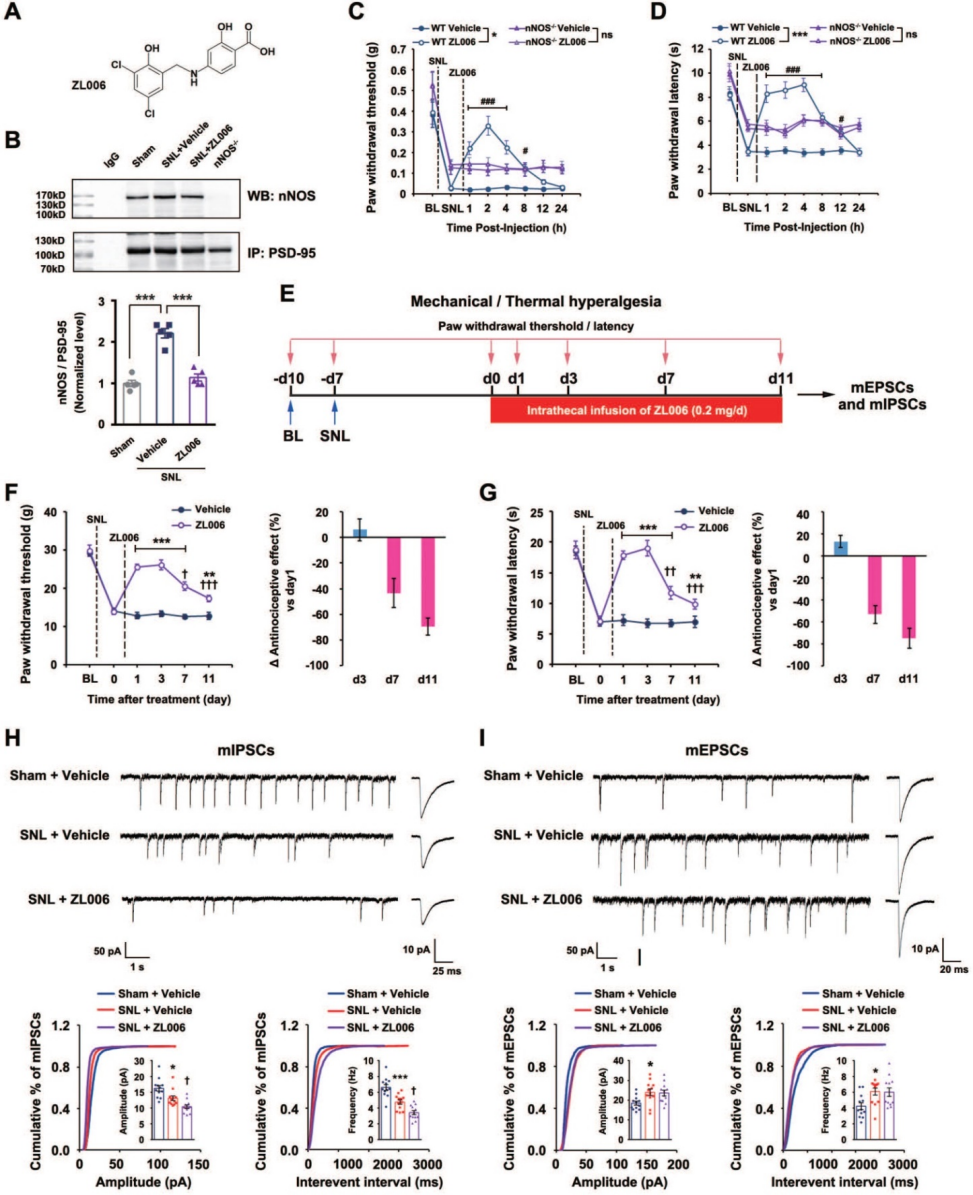
** Chronically uncoupling PSD-95-nNOS exacerbates SNL-induced disinhibition in spinal dorsal horn and leads to analgesic tolerance.** (A) Chemical structure of ZL006. (B) Co-IP showing PSD-95-nNOS complex levels in the spinal dorsal horn of rats subjected to SNL and treated by ZL006 (F (2,12) = 51.066, ^***^p < 0.001, sham vs vehicle, ^***^p < 0.001, vehicle vs ZL006, ANOVA, *n* = 5). WB, western blot; IP, Immunoprecipitation. (C) Pharmacodynamics of ZL006 (i.v., 20 mg/kg) on SNL-induced mechanical hyperalgesia in WT and nNOS^-/-^mice. (*F* (3,34) = 5.829, among groups: ^*^p < 0.05, ZL006 *vs* vehicle; ^###^*p* < 0.001, ^#^*p* = 0.024 *vs* vehicle, repeated measurements ANOVA, WT: n = 9, nNOS^-/-^: n = 10). (D) Pharmacodynamics of ZL006 (i.v., 20mg/kg) on SNL-induced thermal hyperalgesia in WT and nNOS^-/-^mice (*F* (3,32) = 19.554, among groups: ^***^p < 0.001, ZL006 *vs* vehicle; ^###^*p* < 0.001, ^#^*p* = 0.019 *vs* vehicle, repeated measurements ANOVA, n = 9). (E) Design of the experiments for F-I. (F) Left, mechanical hyperalgesia expressed as paw withdrawal threshold of the rats subjected to SNL surgery and treated with ZL006 (0.2 mg/d × 11 days, per animal) or vehicle (*F* (1,22) = 110.00, between groups, ***p < 0.001, **p = 0.001; within group, †*p* = 0.034 (d 7 vs d 1), †††*p* < 0.001 (d 11 vs d 1), repeated measurements ANOVA, n = 12-13). Right, the percent change of thermal threshold for each subject. (G) Left, thermal hyperalgesia expressed as paw withdrawal latency of the rats subjected to SNL surgery and treated with ZL006 (0.2 mg/d ×11 days, per animal) or vehicle, (*F*(1,22) =357.489, between groups, ***p < 0.001, **p = 0.002; within group, ††*p* = 0.004 (d 7 vs d1), †††*p* = 0.0001 (d 11 vs d1), repeated measurements ANOVA, n = 12). Right, the percent change of thermal threshold for each subject. (H-I) Recordings of mIPSCs (H) and mEPSCs (i) from acute spinal slices of the rats undergoing SNL surgery and treated with ZL006 (0.2 mg/d ×11 days, per animal) or vehicle. Upper, representative (left) and averaged (right) traces of mPSCs. Lower, cumulative distribution plots and bar graphs showing amplitude (left) and frequency (right) of mPSCs. In H, for amplitude, *F*(2,33) = 15.384, Sham vs vehicle: *p = 0.020, ZL006 vs vehicle: †*p* = 0.0497; for frequency, *F*(2,33) = 28.059, Sham vs vehicle: ***p = 0.0006, ZL006 vs vehicle: †*p* = 0.014, one-way ANOVA, n = 12). In I, for amplitude, *F*(2,33) = 4.815, Sham vs vehicle: * *p* = 0.032; for frequency, *F*(2,33) = 4.942, Sham vs vehicle: *p = 0.031, one-way ANOVA, n = 12). All data were shown as mean ± SEM.

A growing number of studies have found that ZL006 (Figure 1A), a small molecular compound we developed to treat stroke by disrupting PSD-95-nNOS interaction 15, is effective in different preclinical models assessing pain behavior, attenuating both inflammatory and neuropathic pain in rodents 12, 17, 19, 20. However, our recent study showed that it produces analgesic tolerance after chronic use 21. To further determine whether the antinociceptive effect of ZL006 results from reduced nNOS-PSD-95 association, we measured nNOS-PSD-95 complex level in the SDH of rats and found that segmental spinal nerve ligation (SNL) surgery caused a significant increase in the nNOS-PSD-95 complex and treatment with ZL006 (10 mg/kg, i.v.) reversed the SNL-induced upregulation of PSD-95-nNOS interaction (Figure 1B). Moreover, systemic administration of ZL006 attenuated both mechanical and thermal hyperalgesia markedly in WT but not in nNOS^-/-^ mice (Figure 1C-D) suggesting that ZL006 relieves neuropathic pain through dissociating nNOS-PSD-95. Next, we intrathecally infused ZL006 (0.2 mg/d) for 11 consecutive days using osmotic pump into the vertebral column of rats subjected to SNL and measured mechanical and thermal hyperalgesia (Figure 1E). We found that antinociceptive effect of ZL006 decreased dramatically at d 7 and d 11, compared to d 1 (Figure 1F-G), indicating a remarkable analgesic tolerance. Moreover, the chronic intrathecal infusion of ZL006 aggravated SNL-induced disinhibition of laminae I excitatory neurons in the SDH, as frequency and amplitude of miniature inhibitory postsynaptic currents (mIPSCs) in ZL006-treated rats were significantly lower than that in vehicle-treated rats (Figure 1H). The chronic infusion of ZL006 did not affect SNL-induced increase in miniature excitatory postsynaptic currents (mEPSCs) (Figure 1I). Thus, the prolonged disassociation of PSD-95-nNOS by ZL006 impairs inhibitory synaptic transmission and leads to analgesic tolerance.

### Co-targeting PSD-95-nNOS and α2- and α3-containing GABA_A_Rs avoids central sensitization and analgesic tolerance

**Figure 2:**
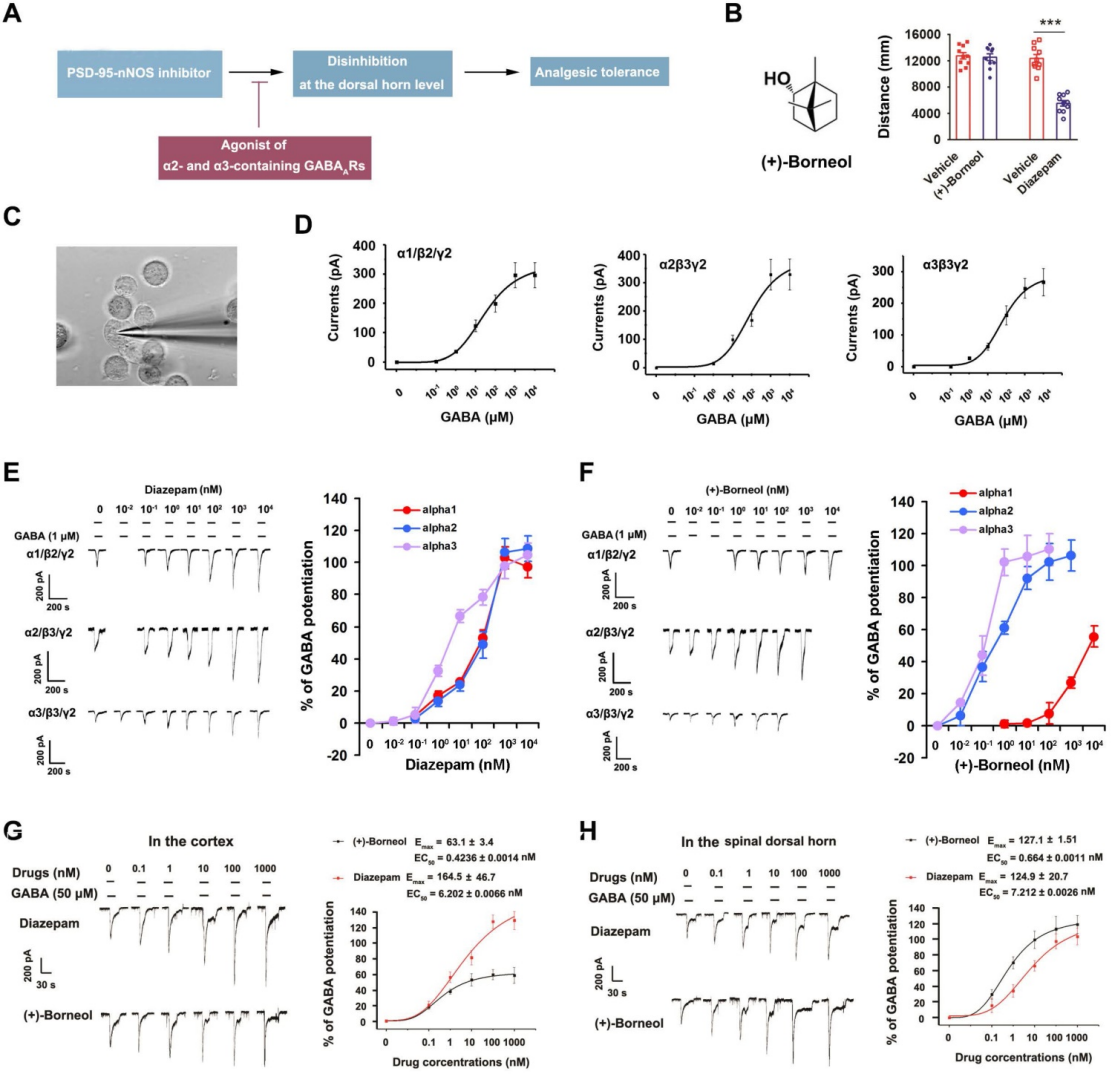
** (+)-Borneol is a positive allosteric modulator of α2- and α3-containing GABAARs without BZDs-associated behavioral effects.** (A) The hypothesis: chronic use of PSD-95-nNOS inhibitor causes analgesic tolerance through the disinhibition of pain neural circuit at spinal level, and α2- and α3-containing GABA_A_Rs agonist reverses this process. (B) Left, chemical structure of (+)-borneol. Right, locomotor activities of the mice treated by (+)-borneol at supramaximal doses (45 mg/kg, i.v.) or diazepam (0.5 mg/kg, i.v.) in the open field test. Locomotor activity was expressed as distance travelled during 5 min after drug injection (*F* (3,36) = 37.33, ^***^p < 0.001, vs vehicle, ANOVA, n = 10). (C) Representative image showing patch-clamp from HEK293 cells transiently expressing GABA_A_Rs (images under 40 × magnification). (D) Dose/response plots of GABA-evoked currents in the HEK293 cells transiently expressing α1β2γ2, α2β3γ2 or α3β3γ2 GABAARs. (E-F) Representative recordings of GABA-evoked currents in the HEK293 cells transiently expressing α1β2γ2, α2β3γ2 or α3β3γ2 GABAARs (left) and dose/response plots (right) of potentiation of GABA-evoked currents by diazepam (E) or (+)-borneol (F), n =8. (G-H) Representative recordings of GABA-evoked currents in putative excitatory neurons (left) of brain cortex (G) and spinal dorsal horn (H) and dose/response plots (right) of potentiation of GABA-evoked currents by diazepam or (+)-borneol, n =12. All data were shown as mean ± SEM.

Based on the findings above, we hypothesized that GABA_A_Rs agonist will neutralize or reverse the disinhibition caused by chronically disrupting PSD-95-nNOS signaling, thereby prevent analgesic tolerance (Figure 2A). Disappointedly, current positive allosteric modulators of GABA_A_Rs, such as benzodiazepines (BZDs), have severe side effects, rendering their use as analgesics problematic 26. GABA_A_Rs are heteropentamers made up from 19 known subunits, including α1-6, β1-3, γ1-3, δ, ε, θ, π, and ρ1-3 24. It is known that α1-containing GABA_A_R in the brain contribute to BZDs-associated sedative effects, while α2- and α3- containing GABA_A_Rs are critical components of spinal pain control 24, 25. Thus, co-targeting PSD-95-nNOS and α2/α3-containing GABA_A_Rs may avoid disinhibition and analgesic tolerance (Figure 2A).

To find a drug potentiating α2- and/or α3-containing GABA_A_Rs selectively, we focused on (+)-borneol, a bicyclic monoterpene alcohol present in the essential oils of numerous medicinal plants 28, 37, 38. The drug has shown antinociceptive effect in both rodents and humans 28, 39 without BZDs-associated behavioral effects (Figure 2B). It has been reported that (+)-borneol can positively modulate α_1_-containing GABA_A_R expressed in Xenopus laevis oocytes at high dose 37. To test the effect of (+)-borneol on other GABA_A_Rs, we compared α1, α2 and α3 selectivity profiles of (+)-borneol and diazepam using electrophysiological experiments in the HEK293 cells transiently expressing recombinant GABA_A_Rs (Figure 2C-D). We found that diazepam had poor selectivity (for α1β2γ2, EC_50_ = 89.7 ± 0.0023 nM, E_max_ = 109.1 ± 18.52%; for α2β3γ2, EC_50_ = 137.74 ± 0.0018 nM, E_max_ = 118.4 ± 16.44%; for α3β3γ2, EC_50_ = 6.14 ± 0.0016 nM, E_max_ = 113.4 ± 8.58%, Figure 2E), while (+)-borneol had very high selectivity for α2- and α3-containing GABA_A_Rs (for α2β3/γ2, EC_50_ = 0.653 ± 0.0017 nM, E_max_ = 121.75 ± 10.97%; for α3β3γ2, EC_50_ = 0.142 ± 0.0013 nM, E_max_ = 108.8 ± 6.05%; for α1/β2/γ2, no effect on GABA-evoked currents as high as 10 μM. Figure 2F). Thus, (+)-borneol is at least 10000 times more selective to α2- or α3-containing GABA_A_R than to α1-containing GABA_A_R. Moreover, (+)-borneol had much smaller effect than diazepam did on brain cortex neurons that mainly express α1-containing GABA_A_R, while they had similar effect on SDH neurons that mainly express α2- and α3-containing GABA_A_Rs (Figure 2G-H).

**Figure 3:**
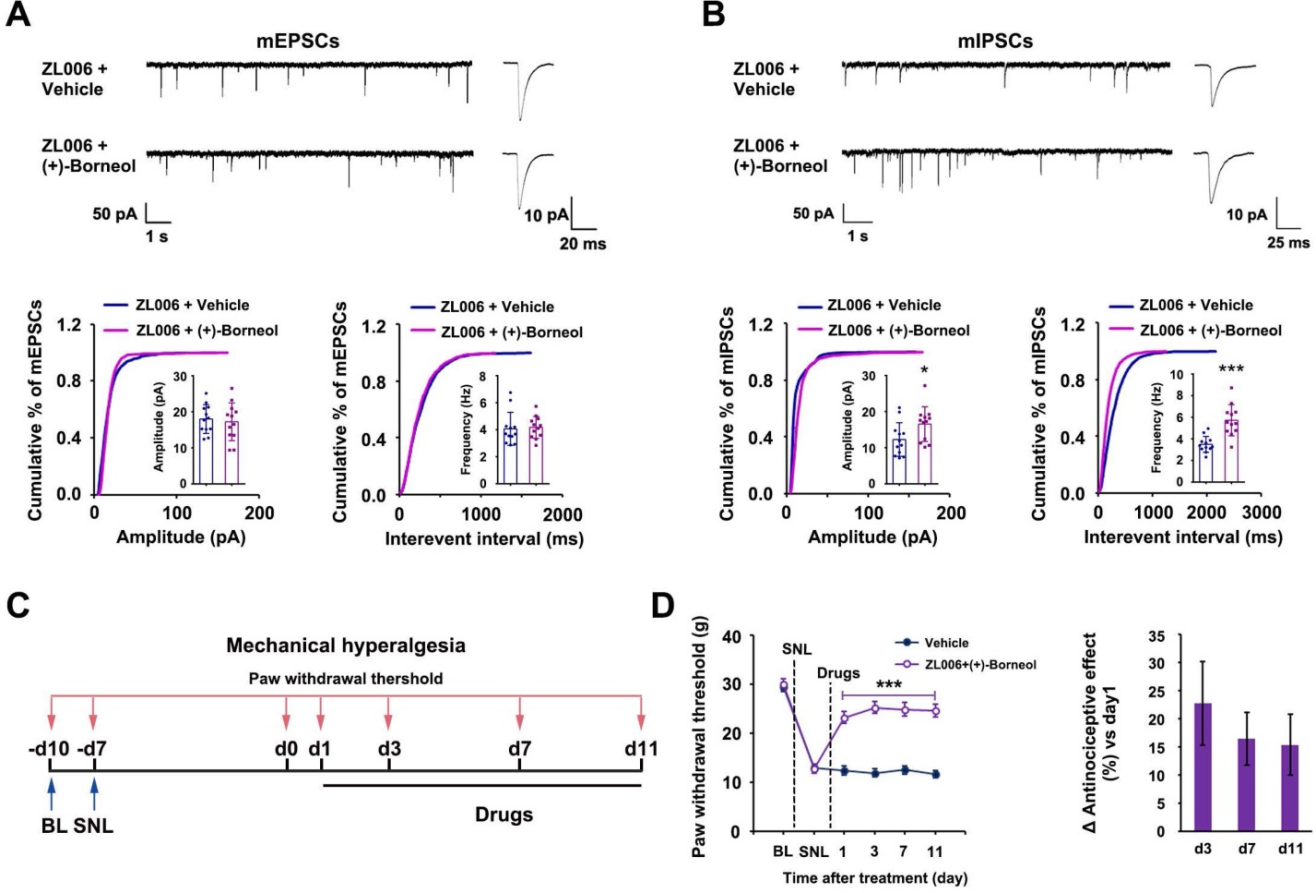
** (+)-Borneol reverses the disinhibition and analgesic tolerance caused by chronically dissociating nNOS-PSD-95.** (A and B) Recordings of mEPSCs (A) and mIPSCs (B) from acute spinal slices of the rats treated with ZL006 or ZL006 with (+)-borneol for 11 consecutive days. Upper, representative (left) and averaged (right) traces of mPSCs. Lower, cumulative distribution plots and bar graphs showing amplitude (left) and frequency (right) of mPSCs (In A, for amplitude, *F* (1,22) = 0.162, *p* = 0.691; for frequency, *F* (1,22) = 0.07, *p* = 0.793, two-tailed *t*-test, n = 12; in B, for amplitude, *F* (1,22) = 4.896, *p* = 0.038; for frequency, *F* (1,22) = 22.788, *p* = 9.127E-05, two-tailed *t*-test, n = 12). (C) Experimental design for D. (D) Left, effect of combination of ZL006 and (+)-borneol on SNL-induced mechanical hyperalgesia (*F* (1,24) = 58.150, ^***^p < 0.001, *vs* vehicle; within cocktail group,* p* > 0.05, repeated measurements ANOVA, n = 13). Right, the percent change of mechanical threshold for each subject. All data were shown as mean ± SEM.

To determine whether (+)-borneol prevents the disinhibition of SDH excitatory neurons caused by chronic use of ZL006, we treated rats with a cocktail consisting of ZL006 (10 mg /kg/d, i.v.) and (+)-borneol (4.7 mg/kg/d, i.v.) for 11 consecutive days. The chronic use of cocktail did not affect mEPSCs but increased mIPSCs frequency and amplitude significantly in putative-excitatory neurons on the laminae I of SDH, compared to ZL006 + vehicle (Figure 3A-B), suggesting that (+)-borneol reverses ZL006-induced disinhibition. Next, we examined whether the chronic use of cocktail avoids analgesic tolerance. We injected the cocktail into the rats subjected to SNL for 11 consecutive days and measured SNL-induced mechanical hyperalgesia, and found that no analgesic tolerance occurred during treatment (Figure 3C-D).

**Figure 4:**
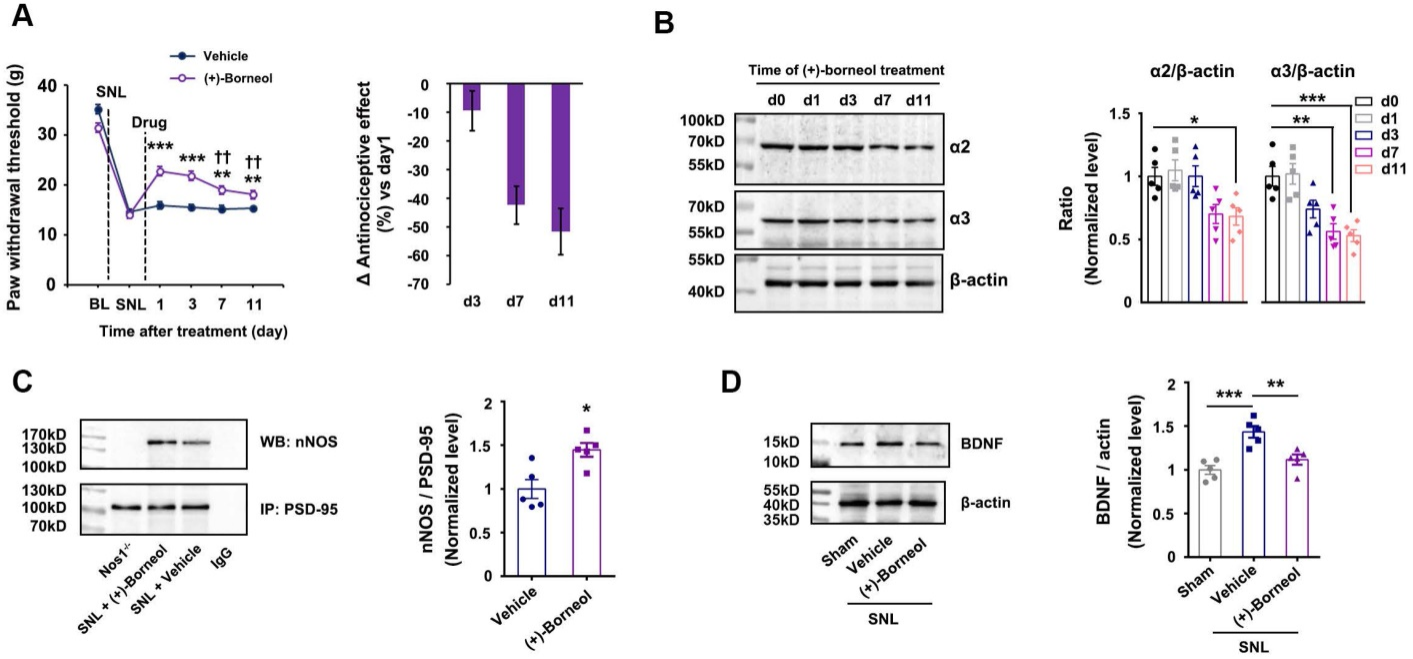
** Effects of (+)-borneol on SNL-induced mechanical hyperalgesia, α2 and α3 subunits of GABA_A_Rs and BDNF expressions and nNOS-PSD-95 interaction in the spinal dorsal horn.** (A) Effect of (+)-borneol (5 mg/kg, i.v.) on SNL-induced mechanical hyperalgesia in rats. (+)-Borneol was used for consecutive 11 days from 7 d after SNL surgery and thresholds were detected at 1 h after drug injection each day (*F* (1,22) = 22.446, between groups: ^**^p = 0.002 (d7), ^**^p = 0.005 (d11), ^***^p < 0.001, *vs* vehicle; within (+)-borneol group: d7 vs d1, ^††^*p* = 0.002; d 11 vs d1, ^††^*p* = 0.003, repeated measurements ANOVA, n = 12). (B) Immunoblots showing α2 and α3 subunits of GABA_A_Rs levels in the spinal dorsal horn of rats treated by (+)-borneol (5 mg/kg/d, i.v., × 11 d). Levels of α2 and α3 were measured at 2 h after each injection (for α2 subunit, *F* (4, 20) = 5.546, ^*^p = 0.025, d0 *vs* d11; for α3 subunit, *F* (4, 20) = 11.479, ^**^p = 0.002, d 0 *vs* d 7; ^***^p = 0.00098, d0 *vs* d11, ANOVA, n = 5). (+)-Borneol was given from 7 days after SNL. (C) PSD-95-nNOS complex levels in the spinal dorsal horn of rats subjected to SNL and treated by (+)-borneol (5 mg/kg/d, i.v., × 3 d, *F*(1,8) = 2.912, ^*^p = 0.0195, two-tailed *t*-test, n = 5). WB, western blot; IP, Immunoprecipitation. Nos1^-/-^, nNOS KO mice, as a negative control. IgG, immunoglobulin G. (D) BDNF levels in the spinal dorsal horn of rats subjected to SNL and treated by (+)-borneol (10 mg/kg, i.v., × 3 d, *F* (2, 12) = 14.508, ^***^p = 0.0007, sham *vs* vehicle; ^**^p = 0.008, vehicle *vs* (+)-borneol, ANOVA, n = 5). All data were shown as mean ± SEM.

Next, we treated the rats subjected to SNL with (+)-borneol (5 mg/kg/d, i.v.) alone for 11 consecutive days and found that the chronic use caused analgesic tolerance development rapidly (Figure 4A), and decreased α2 and α3 subunits of GABA_A_Rs expressions and increased nNOS-PSD-95 interaction in the SDH significantly (Figure 4B, 4C). Therefore, decreased α2 and α3 subunits expressions and increased nNOS-PSD-95 association in the SDH may explain (+)-borneol tolerance. To know how (+)-borneol up-regulates nNOS-PSD-95 interaction, we tested the effect of (+)-borneol on BDNF expression, as GABAergic stimulation can repress BDNF expression in mature neurons 40. It is known that BDNF, as a key mediator in the development of chronic pain, can induce association of PSD-95 with TrkB at excitatory synapses under NMDARs activation, leading to a competitive binding between TrkB and nNOS to PSD-95 41, 42. We found that (+)-borneol significantly decreased SNL-induced BDNF expression in the SDH (Figure 4D). Thus, the (+)-borneol-induced nNOS-PSD-95 association might be due to reduced TrkB-PSD-95 association.

### Dual-target compound ZL006-05 is highly effective against chronic pain without analgesic tolerance and side effects

**Figure 5:**
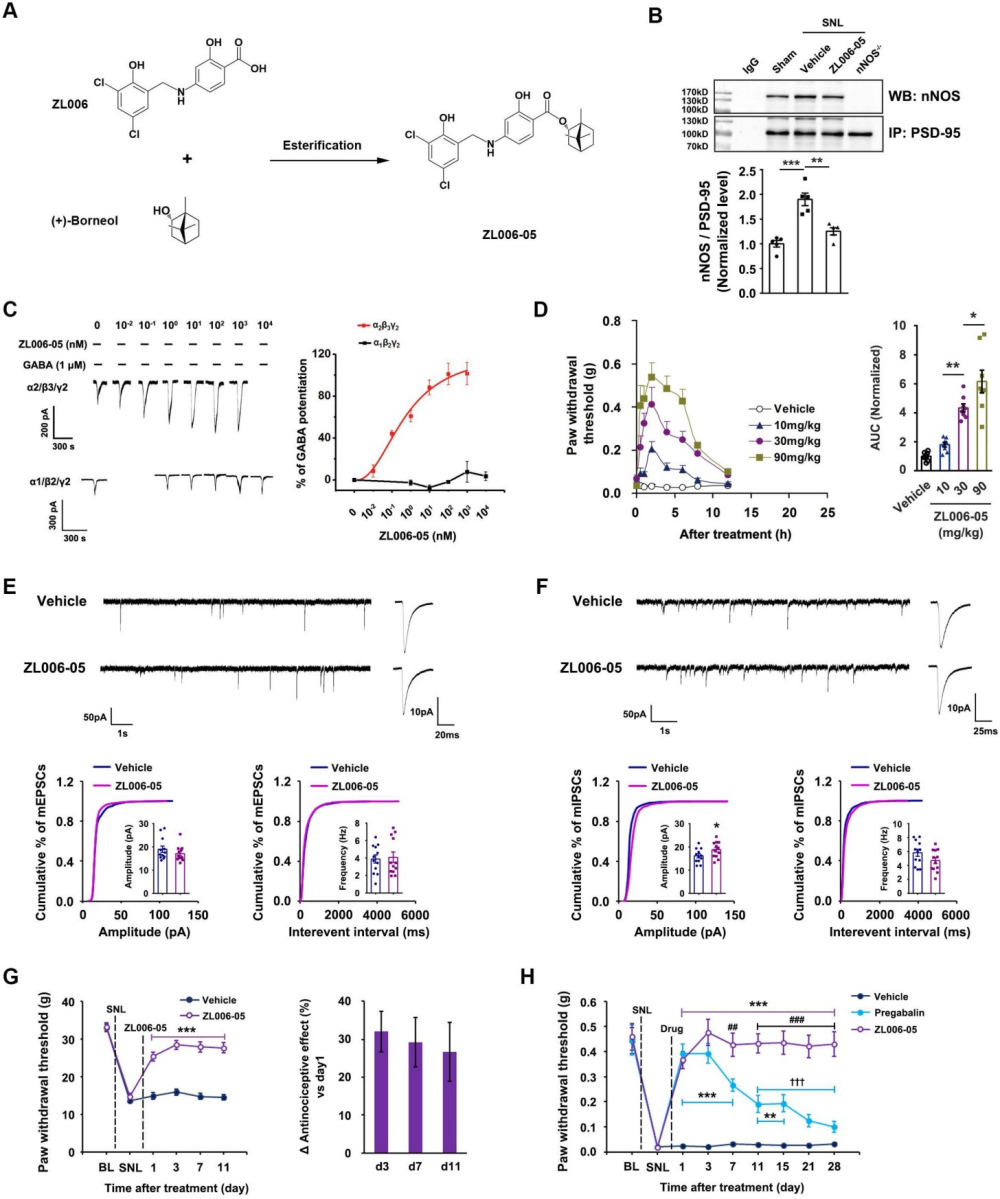
** Chronic use of ZL006-05 avoids central sensitization and analgesic tolerance.** (A) Design of dual-target drug that simultaneously uncouples PSD-95-nNOS and potentiates α2-containing GABA_A_R. (B) Co-IP showing PSD-95-nNOS complex levels in the spinal dorsal horn of rats subjected to SNL and treated by ZL006-05 (*F* (2,12) = 24.787, ^***^p < 0.001, sham vs vehicle; ^**^p = 0.001, vehicle vs ZL006-05, ANOVA, n= 5). WB, western blot; IP, Immunoprecipitation. (C) Representative recordings (left) and dose/response plots (right) of potentiation of GABA-evoked currents by ZL006-05 in the HEK293 cells transiently expressing α1β2γ2 or α2β3γ2 (n = 8). (D) Left, the time courses of SNL-induced mechanical hyperalgesia in mice after treating with ZL006-05 (i.g.). Right, areas under the curve of paw withdrawal threshold 0-12 h after treatment. Paw withdrawal latency response to mechanical stimulation was detected at 0, 0.5, 1, 2, 4, 6, 8, and 12h after drug administration. (*F* (3,28) = 31.64, ^*^p = 0.041, ^**^p = 0.003, ANOVA, n =8). (E, F) Recordings of mEPSCs and mIPSCs from acute spinal slices of the rats that received intrathecal infusion of ZL006-05 (0.3 mg/d, per animal, intrathecal infusion using osmotic pump, ×11 d). Upper, representative (left) and averaged (right) traces of mPSCs. Lower, cumulative distribution plots and bar graphs showing amplitude (left) and frequency (right) of mPSCs (In E, for amplitude, *F* (1,22) = 1.136, *p* = 0.268; for frequency, *F* (1,22) = 0.2604, *p* = 0.797; in F, for amplitude, *F* (1,22) = 2.005, ^*^p = 0.0119; for frequency, *F* (1,22) = 1.640, *p* = 0.239; two-tailed *t*-test, n = 12). (G) Left, effect of ZL006-05 (60 mg/kg/d, i.g., ×11 d) on SNL-induced mechanical hyperalgesia in rats (*F* (1,24) = 89.591, ^***^p < 0.001, *vs* vehicle, n = 13). Right, the percent change of thermal threshold for each subject. (H) Effects of ZL006-05 (60 mg/kg/d, i.g., × 28 d) and pregabalin (30 mg/kg/d, i.g., × 28 d) on SNL-induced mechanical hyperalgesia in mice (*F* (2,33) = 53.208, among groups: ^**^p < 0.01, ^***^p < 0.001, *vs* vehicle; ^##^*p* = 0.002, ^###^*p* < 0.001, ZL006-05 *vs* pregabalin; within group: ^†††^*p* < 0.001, *vs* d1, n = 12). Repeated measurements ANOVA in G and H. All data were shown as mean ± SEM

Next, we designed and synthesized a dual-target compound ZL006-05 that linked ZL006 and (+)-borneol through an ester bond (Figure 5A). To determine whether ZL006-05 disrupts PSD-95-nNOS interaction and potentiates α2-containing GABA_A_Rs selectively, we examined its effects on PSD-95-nNOS interactin in the SDH, and electrophysiological properties in the HEK293 cells transiently expressing recombinant GABA_A_Rs. As expected, ZL006-05 not only abolished the SNL-induced increase in PSD-95-nNOS complex in the SDH (Figure 5B) but also showed a very high affinity to α2-containing GABA_A_R (Figure 5C and Figure S1). Interestingly, intragastric administration (i.g.) of ZL006-05 reduced SNL-induced mechanical hyperalgesia and thermal pain and complete Freund's adjuvant (CFA)-induced inflammatory pain (Figure 5D, Figure S2). Because of rigid structure of (+)-borneol and stereo-hindrance effect, we predict that ZL006-05 will not be hydrolyzed into ZL006 and (+)-borneol *in vivo*. The enzymatic hydrolysates may have discordant pharmacokinetics. To address this, we measured metabolites of ZL006-05 in blood. As expected, neither ZL006 nor (+)-borneol was detectable in the blood after ZL006-05 administration (i.v. or i.g.), and parent drug was main metabolite (Figure S3).

To test whether chronic use of ZL006-05 affects synaptic transmission, we treated rats with ZL006-05 by intrathecal infusion using osmotic pump for 11 consecutive days and measured mIPSCs and mEPSCs in putative excitatory neurons on the laminae I of dorsal horn in acute spinal slices. Interestingly, the chronic use of ZL006-05 significantly increased mIPSCs amplitude, without affecting mEPSCs (Figure 5E-F), which was totally different from the chronic use of ZL006 (Figure 1). In addition, the chronic use of ZL006-05 (i.g.) did not produce analgesic tolerance (Figure 5G). To further strengthen the finding, we treated the mice subjected to SNL surgery with ZL006-05 or pregabalin, a representative of gabapentinoids that are currently recommended as the first-line choices for neuropathic pain. ZL006-05 maintained its antinociceptive effect during 28 days treatment, showing a long-term relief of neuropathic pain without analgesic tolerance, while pregabalin caused a severe analgesic tolerance (Figure 5H).

**Figure 6:**
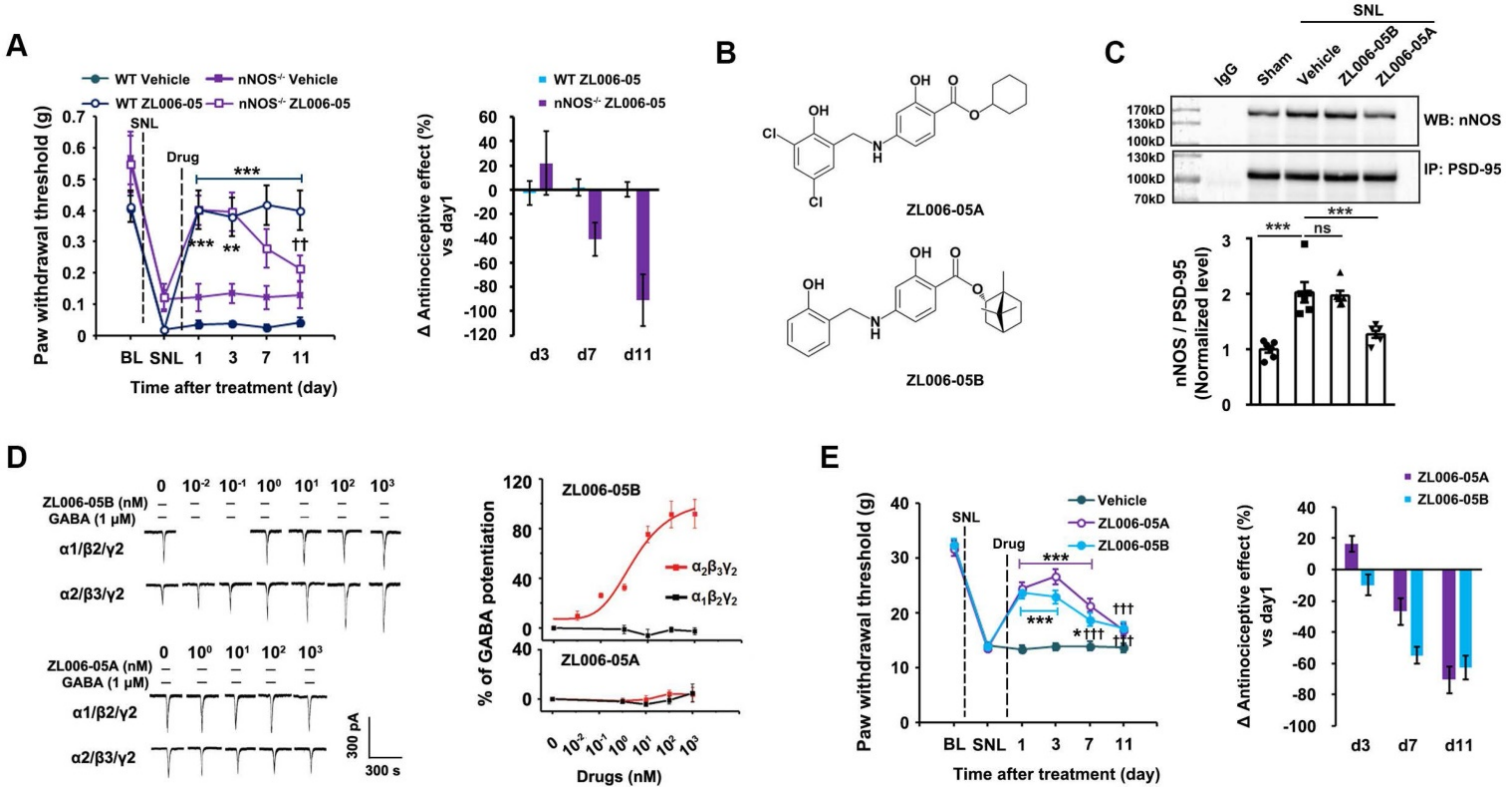
** Dual-target is necessary for preventing analgesic tolerance.** (A) Left, effect of ZL006-05 on SNL-induced mechanical hyperalgesia in WT and nNOS KO mice (*F* (3,44) = 8.277, among groups: ^***^p < 0.001, ^**^p < 0.01, ZL006-05 *vs* vehicle; within group: ^††^*p* < 0.01, d11 *vs* d1, n = 12). Right, the percent change of thermal threshold for each subject. (B) Chemical structures of analogues of ZL006-05. (C) Co-IP showing PSD-95-nNOS complex levels in the spinal dorsal horn of rats subjected to SNL and treated by ZL006-05A or ZL006-05B (*F* (3,20)= 21.086, ^***^p < 0.001, ANOVA, n= 6). (D) Representative recordings (left) and dose/response plots (right) of potentiation of GABA-evoked currents by ZL006-05A or ZL006-05B in the HEK293 cells transiently expressing α1β2γ2 or α2β3γ2 (n =8). For α2β3γ2 in the ZL006-05B-treated cells, EC_50_ = 2.834 ± 0.0033 nM, E_max_ = 104.43 ± 21.93%. (E) Left, effect of ZL006-05A or ZL006-05B on SNL-induced mechanical hyperalgesia in rats (*F* (2,39) = 19.730, among groups: ^*^p = 0.015, ^***^p < 0.001, *vs* vehicle; within group: ^†††^*p* < 0.001, *vs* d1, n = 14). Right, the percent change of thermal threshold for each subject. Repeated measurements ANOVA in A and E. Animals were treated with drugs (60 mg/kg/d, i.g.) for 3 d in C, and for 11 d in A and E. All data were shown as mean ± SEM.

To determine whether the dual-target is necessary for preventing analgesic tolerance, we observed the antinociceptive effect of ZL006-05 in the nNOS^-/-^ and WT mice subjected to SNL during 11 days treatment. ZL006-05 caused a remarkable analgesic tolerance in nNOS^-/-^ mice but not in WT mice (Figure 6A), indicating a reappearance of analgesic tolerance after the deletion of PSD-95-nNOS target. To further determine whether PSD-95-nNOS disassociation and α2-containing GABA_A_R potentiation are both necessary for preventing analgesic tolerance, we modified the structure of ZL006-05 to delete its ability to target PSD-95-nNOS or GABA_A_Rs (Figure 6B). Both ZL006-05A, one ZL006-05 analogue without ability to target α2-containing GABA_A_R, and ZL006-05B, another ZL006-05 analogue without ability to target PSD-95-nNOS (Figure 6B-D), caused substantial analgesic tolerance (Figure 6E). Together, dissociation of PSD-95-nNOS or potentiation of α2-containing GABA_A_R alone can't prevent analgesic tolerance development.

**Figure 7:**
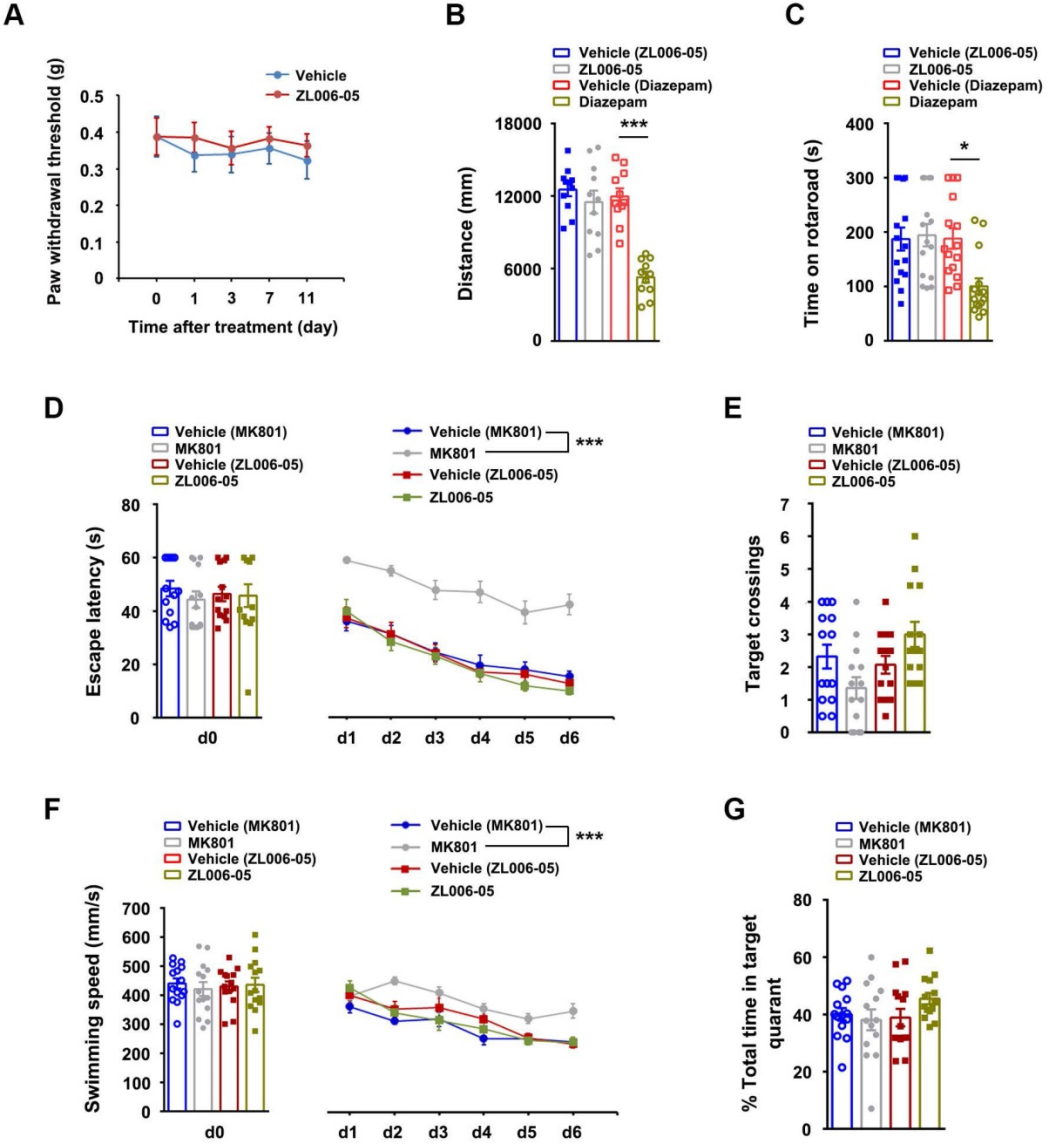
** ZL006-05 has no unwanted side effects.** (A) Mechanical hyperalgesia in rats under physiological conditions. ZL006-05 (90 mg/kg/d, i.g., × 11 d) was administrated at 2 h before each measurement (*F* (1,28) = 0.327, *p* > 0.05, between groups or within group, repeated measurements ANOVA, n = 15). (B) Locomotor activity was expressed as distance travelled during 5 min and was measured at 1 h after drug injections (ZL006-05, 60 mg/kg, i.v.; diazepam, 0.5 mg/kg, i.v.) in the Open Field in mice (*F* (3,40) = 17.69, ^***^p < 0.001, ANOVA, n = 11). (C) Motor coordination was expressed as time spent on the accelerating rotarod in rats and was measured at 30 min after injection of ZL006-05 (60 mg/kg, i.v.) or diazepam (0.5 mg/kg, i.v.) (*F* (3,56) = 5.61, ^*^p = 0.019, ANOVA, n = 15). (D) The latency of rats finding a hidden platform (for d0, *F* (3,52) = 0.31, *p* > 0.05, between groups, ANOVA; for d1-d6, *F* (3,52) = 60.416,^***^p < 0.001, repeated measurements ANOVA, n = 14,). (E) The number of crossings of the platform location (*p* > 0.05, between groups, ANOVA, n= 14). (F) Swimming speed of rats during the period indicated in the Morris water maze (for d0, *F* (3,52) = 0.15, *p* > 0.05, between groups, ANOVA; for d1-d6, *F* (3,52) = 11.372, ^***^p < 0.001, repeated measurements ANOVA, n= 14). ZL006-05 (90 mg/kg/d, i.g.) and MK801 (0.25 mg/kg/d, i.v.) was administrated at 2 and 1 h before first trial everyday respectively. (G) Time spent in the target quadrant in the probe test on day 7 (*p* > 0.05, between groups, ANOVA, n = 14). All data were shown as mean ± SEM.

To test whether the lack of ZL006-05 tolerance is due to the alteration of basic pain threshold under normal conditions, we measured mechanical nociceptive thresholds in normal rats before drug and at 2 h after the injection of ZL006-05 every day. Chronic use of ZL006-05 for 11 consecutive days did not change the basic threshold (Figure 7A). Next, we investigated typical side effects mediated by GABA_A_Rs (sedation and motor impairment) and NMDARs (cognitive impairment). Diazepam reduced the locomotor activity of mice in the open-field test and shortened the time for rats to fall off an accelerating rotarod significantly, while ZL006-05 had no effect (Figure 7B-C). In the Morris water maze task, NMDAR antagonist MK801 but not ZL006-05 caused significantly impaired spatial memory (Figure 7D-G). Thus, ZL006-05 has no unwanted side effects associated with BZDs and NMDARs antagonists.

## Discussion

**Figure 8:**
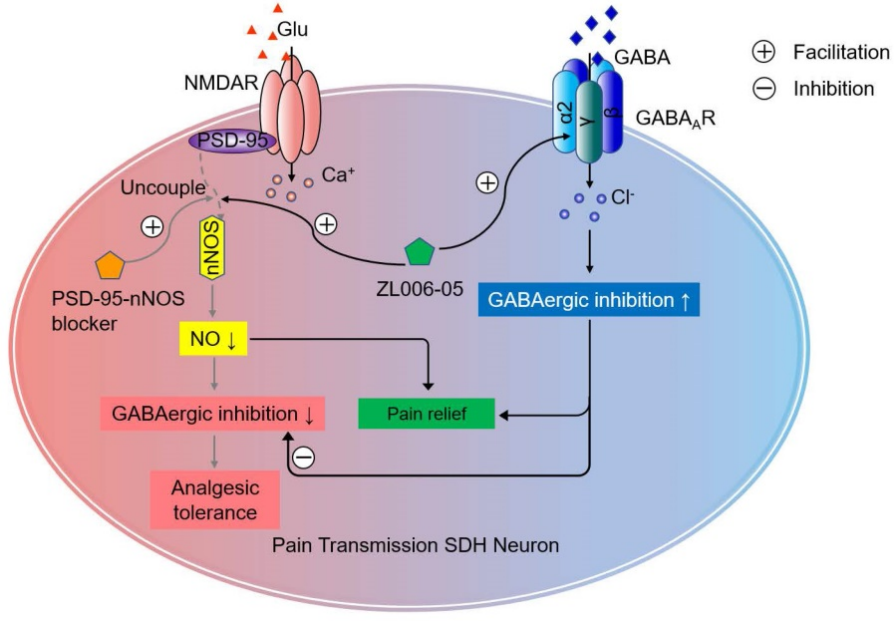
Schematic illustration of the analgesic mechanism of the dual-target pain killer ZL006-05. Chronic use of PSD-95-nNOS blocker develops analgesic tolerance due to NO reduction-induced GABAergic disinhibition, while ZL006-05 can avoid the analgesic tolerance by co-targeting α2-containing GABAARs and nNOS-PSD-95 interaction.

Chronic neuropathic pain is a challenging clinical problem because the pain is often severe, persistent and disabling 7, 43. Directly blocking NMDARs or its downstream signaling nNOS-PSD-95 can cause analgesic tolerance in neuropathic pain due to GABAergic disinhibition 21. In the present study, we found that (+)-borneol selectively potentiated α2- and α3-containing GABA_A_Rs and prevented the disinhibition of SDH excitatory neurons and analgesic tolerance caused by nNOS-PSD-95 blocker. Based on the finding, we designed a dual-target compound ZL006-05 by linking ZL006 and (+)-borneol through an ester bond, and found that it blocked nNOS-PSD-95 interaction and selectively potentiated α2-containing GABA_A_R, a special subtype for spinal pain control, and more importantly, did not produce analgesic tolerance and unwanted side effects, indicating a potential for clinical translation (Figure 8).

Our previous and current studies show that both NMDAR antagonist MK801 and nNOS-PSD-95 disruptor ZL006 cause analgesic tolerance after chronic use, mainly due to GABAergic disinhibition. 21. The GABAergic disinhibition also occurs after chronic morphine treatment 44. Besides, gabapentin, a drug currently recommended as the first-line choices for neuropathic pain, causes analgesic tolerance in poststroke pain after continuous use for 2 weeks 45. Actually, it has been widely accepted that impaired inhibitory control in the pain neural circuits of SDH is responsible for various chronic pain forms. Owing to impaired inhibitory transmission in the SDH after neuropathic injury, the feed-forward disynaptic inhibition of Aβ-fiber is removed, which drives mechanical allodynia 6. Especially, GABAergic inhibition normally separates superficial laminae I and II, to which peripheral noxious information is transmitted, from laminae III and IV by suppressing the activity of pre-existing excitatory synaptic circuits 7, 22, 46. Thus, the attenuation of GABA_A_Rs-mediated inhibitory synaptic transmission undoubtedly plays a key role in the aberrant processing of pain information in the dorsal horn.

Most GABA_A_Rs in the brain and spinal cord are heteropentameric ion channels composed of two α, two β and one γ2 subunit 26. The biding site for BDZs is formed by one of α subunits, including α1, α2, α3 and α5 24. Sedative actions of BDZs occur through α1GABA_A_R 47. Impaired motor coordination involves α1GAB_A_Rs and/or α3GABA_A_Rs 26. Cognitive impairment likely originates from α5GABA_A_Rs 48. Interestingly, almost all the antinociceptive efficacy of BDZs comes from the activation of α2GABA_A_Rs 25, 26. Highly selective α2GABA_A_Rs agonists should therefore have the best benefit-risk ratio. In this study, we found that (+)-borneol is a positive allosteric modulator of GABA_A_Rs with high selectivity of GABA_A_Rs containing α2/α3 subunits. However, analgesic tolerance and its potent effect on α3GABA_A_Rs may affect clinical translation of (+)-borneol. Interestingly, ZL006-05 we designed is a relatively pure α2GABA_A_Rs agonist, as it has very high efficacy and potency on α2GABA_A_Rs and low efficacy on α3GABA_A_R and α5GABA_A_R, and does not potentiate α1GABA_A_R.

Chronic pain is often complicated by general anxiety and depressed mood 49, 50. Inhibitors of nNOS-PSD-95 interaction, such as ZL006, have antidepressant-like properties 51 and attenuate negative affective component of pain 52. GABA_A_Rs agonists are the most commonly prescribed anxiolytics and α2-containing GABA_A_Rs have physiological anti-depressant-like role 5. Thus, by co-targeting PSD-95-nNOS interaction and α2-containning GABA_A_R, ZL006-05 may not only produce analgesic effect but also relieve negative emotions of pain. Moreover, both excessive nNOS-PSD-95 association and GABA_A_Rs dysfunction contribute to excitotoxity in the brain 15, 53. Neurotoxicity is widely accepted to be a common mechanism for stroke and neurodegenerative diseases 54. Thus, our findings could have wider implications beyond neuropathic pain.

## Abbreviations

NMDAR: N-methyl-D-aspartate receptor
SDH: spinal dorsal horn
PSD-95: postsynaptic density-95
nNOS: neuronal nitric oxide synthase
GABA_A_Rs: γ-aminobutyric acid type A receptors
BDNF: brain-derived neurotrophic factor
mIPSCs: miniature inhibitory postsynaptic currents
mEPSCs: miniature excitatory postsynaptic currents
SNL: spinal nerve ligation
CFA: complete Freund's adjuvant
