# Supplementary material for: A pain killer without analgesic tolerance designed by co-targeting PSD-95-nNOS interaction and α2-containning GABAARs
Source: Theranostics. 2021 Apr 3;11(12):5970–85. doi: 10.7150/thno.58364 (PMC8058733; doi:10.7150/thno.58364)
Supplement: Supplementary file 1 — Supplementary figures. [file thnov11p5970s1.pdf]

Supplementary Material

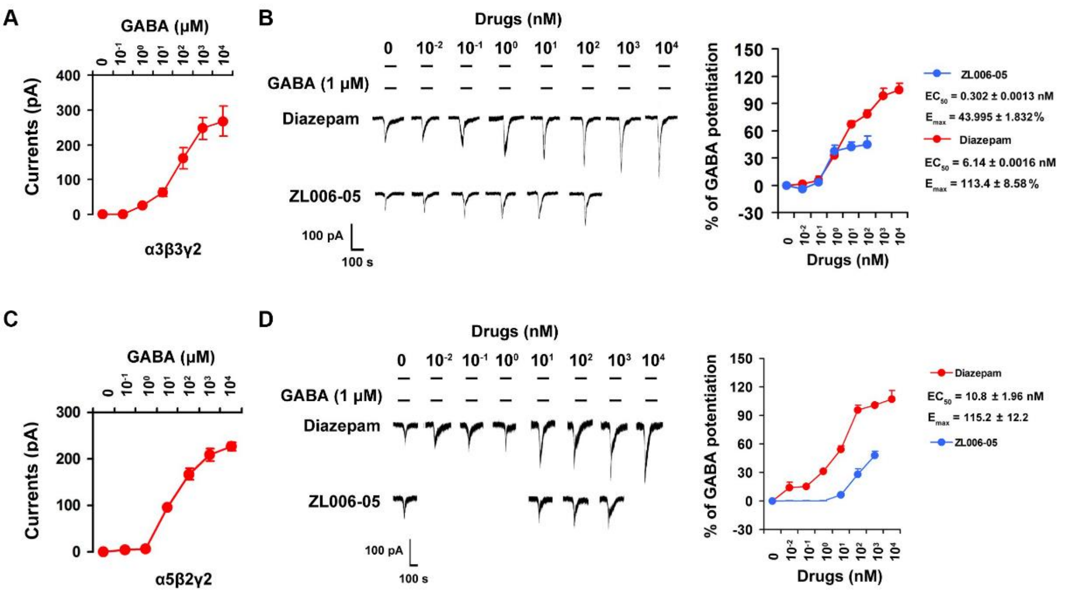

Supplementary Figure 1. Effects of Diazepam and ZL006-05 on α3- and α5-containing GABAARs.

(**A**) Dose/response plots of GABA-evoked currents in the HEK293 cells transiently expressing α3β3γ2 $GABA_A$Rs. (**B**) Representative recordings of GABA-evoked currents in the HEK293 cells transiently expressing α3β3γ2 $GABA_A$Rs (left) and Dose/response plots (right) of potentiation of GABA-evoked currents by diazepam or ZL006-05 (n = 8). (**C**) Dose/response plots of GABA-evoked currents in the HEK293 cells transiently expressing α5β3γ2 $GABA_A$Rs. (**D**) Representative recordings of GABA-evoked currents in the HEK293 cells transiently expressing α5β3γ2 $GABA_A$Rs (left) and dose/response plots (right) of potentiation of GABA-evoked currents by diazepam or ZL006-05 (n = 8). E_{max} and EC_{50} were not estimated because of the solubility limit of ZL006-05. All data are shown as mean ± SEM.

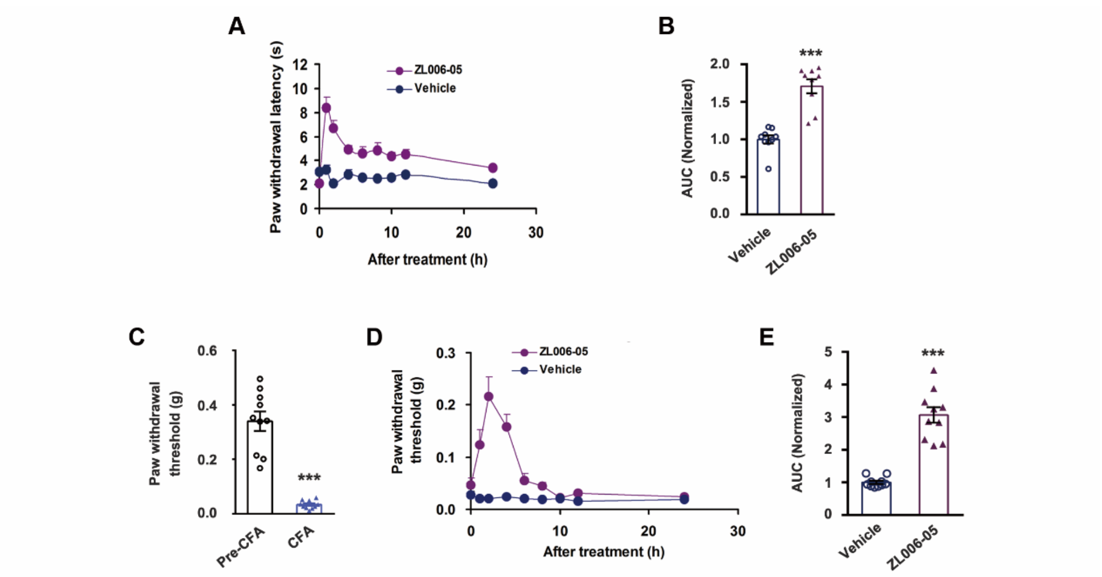

Supplementary Figure 2. Effect of ZL006-05 on SNL-induced thermal hyperalgesia and CFA-induced inflammatory pain in mice.

(**A**) Time course of SNL-induced thermal hyperalgesia after treating with ZL006-05 (60 mg/kg, i.g.) in mice. Paw withdrawal latency response to thermal stimulation was detected at 0, 1, 2, 4, 6, 8, 10, 12 and 24 h after drug administration. (**B**) Areas under the curve of paw withdrawal latency 0-24 h after treatment calculated from (**A**) (F (1,16) = 43.07, $^{***}p < 0.001$, two-tailed t-test, n = 9). (**C**) Paw withdrawal threshold response to mechanical stimulation before and 24 h after CFA injection in mice (F (1,18) = 72.12, $^{***}p < 0.001$, two-tailed t-test, n = 10). (**D**) Time course of ZL006-05 on CFA-induced inflammatory pain. (**E**) Areas under the curve of paw withdrawal latency 0-24 h after treatment calculated from (**D**) (F (1,18) = 71.73, $^{***}p <0.001$, two-tailed t-test, n = 10). ZL006-05 (60 mg/kg, i.g.) was given 24 h after CFA injection and paw withdrawal threshold response to mechanical stimulation was detected at 0, 1, 2, 4, 6, 8, 10, 12 and 24 h after drug administration. All data are shown as mean ± SEM.

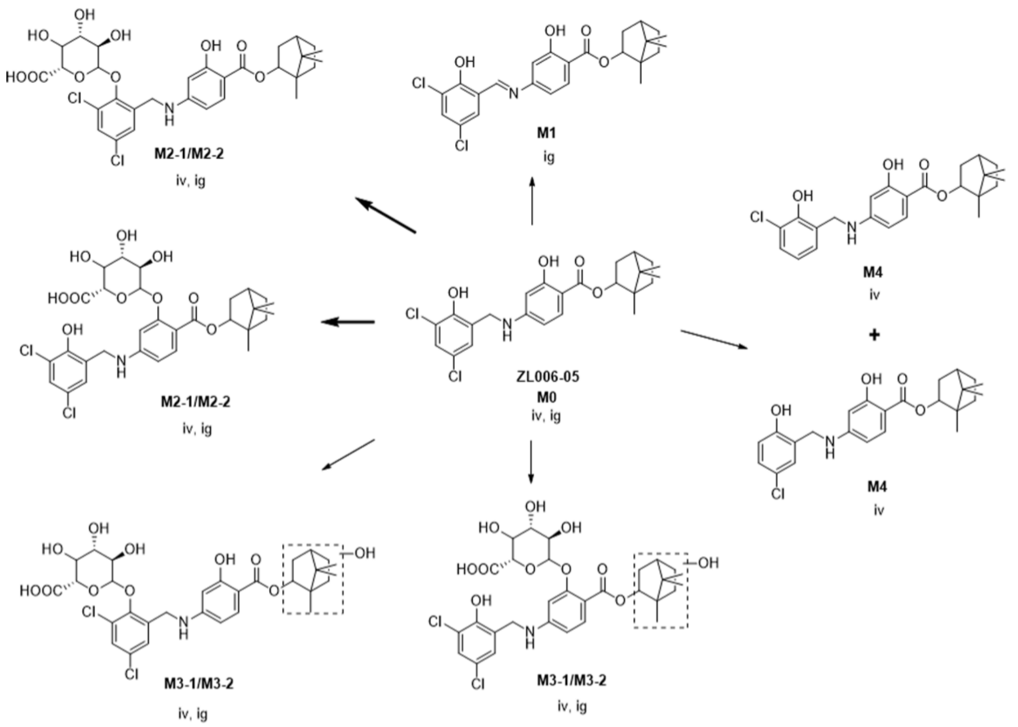

Supplementary Figure 3. Proposed metabolic pathways of ZL006-05 in rats.

Three metabolites except parent drug were found in plasma after intravenous injection of ZL006-05, including M2-2 (glucuronide conjunction), M3-2 (Mono-oxidation & glucuronide conjunction) and M4 (dechlorination reduction); five metabolites except parent drug were found in plasma after oral administration of ZL006-05, including M1 (Oxidation by de-H2), M2-1 and M2-2 (glucuronide conjunction), M3-1 and M3-2 (Mono-oxidation & glucuronide conjunction). Based on the chromatogram of UPLC-UV, the main metabolite was parent drug (M0) in plasma after iv dosing, the main metabolite were parent drug (M0) and glucuronide conjunction (M2-2) in plasma after oral dosing, the proposed metabolic pathways of ZL006-05 in blood were summarized. i.v.: intravenous injection; i.g.: oral administration.
